# Editorial: Neuroimaging and informatics for successful aging

**DOI:** 10.3389/fnhum.2022.976524

**Published:** 2022-07-28

**Authors:** Toshiharu Nakai, Eric Tatt Wei Ho, Henning Müller, Fanpei G. Yang, Hanna Lu

**Affiliations:** ^1^Institute of NeuroImaging and Informatics, Obu, Japan; ^2^Department of Dental Radiology, Graduate School of Dentistry, Osaka University, Suita, Japan; ^3^Center for Intelligent Signal and Imaging Research, Universiti Teknologi Petronas, Perak, Malaysia; ^4^Institute of Information Systems, University of Applied Sciences and Arts of Western Switzerland HES-SO Valais-Wallis, Sierre, Switzerland; ^5^Département de Radiologie et Informatique Médicale, Université de Genève, Geneva, Switzerland; ^6^Department of Foreign Languages and Literature, National Tsing Hua University, Hsinchu, Taiwan; ^7^Center for Cognition and Mind Sciences, National Tsing Hua University, Hsinchu, Taiwan; ^8^Department of Psychiatry, The Chinese University of Hong Kong, Shatin, Hong Kong SAR, China

**Keywords:** neuroinformatics, older adults, aging, neuroimaging, cognitive intervention, cohort study, neurotechnology, BrainConnects

Aging of adults usually involves cognitive decline due to various neuronal pathologies and neurophyonsiological changes, resulting in the so-called “geriatric syndrome” in aging. This puts older adults beyond a certain age at a high risk of getting lost and of falling due to declined attention levels, memorization, visuospatial processing and judgments. In this perspective, the increase in life expectancy around the world must be accompanied by health maintenance, social and intellectual participation and individual security toward “successful aging” that aims at limiting some of the negative effects and risks. In order to achieve a better quality of life, various social programs promoting physical activities for older adults are widely organized depending on the social backgrounds and cultures in each country. However, the outcomes of cognitive and physical interventions have been controversial among behavioral studies because of instability and reduced capacity of cognitive functions under progressive cognitive decline as well as difficulty of methodological standardization. Since physical and cognitive intervention protocols are designed based on the popularity of the activity and physical potential of the participants, the response to the interventions may vary among individuals. This renders objective evaluation very complex and prone to evaluation errors due to the high intra-variability reflecting individual differences and often small effect sizes in studies. Thus, we need to develop an objective approach to extract and classify important factors to maintain to support cognitive function and physical activity. This should include not only structured data but should really mix imaging data on neural activities and decline as well as structured data and possibly other measurements. The importance is to find and analyze the links between the data systematically.

In this respect, we recognize that successful aging strongly depends on successful brain aging, and functional and structural neuroimaging are excellent tools to objectively evaluate and (quantitatively) measure specific factors such as plastic change, i.e., compensation, reorganization, and remodeling of aging brains undergoing cognitive and physical decline. Integration of different types of information, such as fMRI, DTI, EEG, or NIRS, as well as neurofeedback from performance or physiological monitoring to optimize learning can enhance the precision. Larger studies with quantitative measures could also really advance this field, as well as inclusion of other factors that can have an influence on cognitive decline, for example behavioral changes (smoking, alcohol, sports, …). The goal of this Research Topic is to explore the role of functional and structural neuroimaging together with neuroinformatics and neurotechnologies to reach “successful brain aging” and further develop an emerging and multidisciplinary field of “aging neuroinformatics.”

This Research Topic, “*Neuroimaging and Informatics for Successful Aging*,” is a result of a long standing international annual research workshop called BrainConnects that started in 2014. The 7th annual meeting, BrainConnects 2020, was transformed into a special issue in Frontiers to overcome the on-going SARS-CoV-2 pandemic. The main goal of this scientific collaboration is to develop and exchange methods and technologies for delaying and modulating neurocognitive changes due to aging that can help enhance and accelerate the projects promoted by the participants from various countries. Our annual interdisciplinary workshop provides an opportunity to introduce and review the ongoing investigations of the dynamics of neuronal circuits in response to cognitive interventions, as well as to promote collaboration among the participating research groups and to nurture young investigators.

Sixteen papers including 3 reviews by 115 authors from 11 countries were judged by 39 reviewers from 18 countries and published across 4 journals in this Research Topic. The study type of the papers can be summarized as the following; four cohort studies evaluated the dependency of a resting state network (RSN) on aging as a biomarker of cognitive performance (Maesawa et al.; Watanabe et al.) and morphologic change (Dominguez et al.; Md Ashraf et al.). Two papers used blood samples to investigate the relationship between plasma amyloid concentration and PET findings (Huang et al.) or biochemical data and brain activation detected by fMRI (You et al.). As neurophysiological studies, the age-related changes of basal ganglia in motor control were explored (Rodorigez-Sabata et al.) and EEG characteristics of Alzheimer disease were reviewed (Yu et al.). As a cellular level approach, an automata model of the aging brain was proposed (Ramos et al.). Brain-wide measures of good cognition in healthy aged brains serve as a potential target for a variety of healthy aging interventions. The RSN patterns identified by Maesawa et al. could be especially meaningful for developing neurofeedback interventions whereas the cortical thickness parameters obtained by Dominguez et al. are relevant to lifestyle-related interventions such as cognitive activities and nutrition.

Six papers reported the effects of cognitive/physical interventions, such as mindful training (Sevinc et al.), verbal training (Fong et al.; Yang et al.; Yoske et al.), musical training (Yamashita, Ohsawa, et al.) or physical training (Yamashita, Suzuki, et al.) on behavioral and neuroimaging outcome and their correlations. The concern regarding verbal training as a method of intervention for older adults may come from its familiarity with the subjects, raising the motivation to train, since languages have an essential role in social participation and human relationships. The use of verbal training as a method of intervention has shown to improve network connectivity and enhance frontal-temporal network activity. Neurotechnology is emerging as an alternative therapy to behavioral interventions and here, state-of-art research into the applications of deep brain stimulation to ameliorate the symptoms of neurodegenerative disease in aging was reviewed by Silverio and Silverio with an emphasis on the electronic circuit technologies required to implement the therapy.

To summarize, these papers span four thematic areas : (i) characterizing the neurodegenerative aspects of aging on the brain (Md Ashraf et al.; Huang et al.; Ramos et al.; Rodriguez-Sabate et al.; Watanabe et al.; Yu et al.; You et al.) (ii) measures of healthy aging in the brain (Maesawa et al.; Dominguez et al.) (iii) evidence of behavioral interventions toward promoting healthy aging in the brain (Fong et al.; Sevinc et al.; Yang et al.; Yeske et al.; Yamashita, Ohsawa, et al.; Yamashita, Suzuki, et al.) and (iv) prospects for a neurotechnology-based therapy to address aspects of aging-linked neurodegenerative disease (Silverio et al.). The papers in area (i) are mostly from clinical side, however, two papers have strong background of engineering science (Md Ashraf et al.; Ramos et al.). Area (ii) is cohort study, studies in area (iii) are approaches from cognitive neuroscience, and area (iv) is engineering science.

The collection of papers in this Research Topic originated from several continents, demonstrating the strong commitment of BrainConnects to link scientists from various disciplines to continue our scientific discourse with a larger community interested in the neurobiology of aging and ways to influence it. We hope that these research articles will share our excitement and enthusiasm for further research collaborations and discussions amongst our readership. We aim to continue research investigations that will bring about meaningful outcomes to answer some of the pressing questions related to cognitive and mental health in aging. Together, we have the common goals of using neurotechnologies and informatics based on solid data to understand and develop innovative methods for successful aging. Expertise in neuroinformatics, such as computational modeling and simulation, data structuring and management, signal recording and processing, atlasing, and ontologies will further evolve aging research to find solutions for the limitations as discussed in each article in this Research Topic.

## Author contributions

All authors listed have made a substantial, direct, and intellectual contribution to the work and approved it for publication.

## Conflict of interest

The authors declare that the research was conducted in the absence of any commercial or financial relationships that could be construed as a potential conflict of interest.

## Publisher's note

All claims expressed in this article are solely those of the authors and do not necessarily represent those of their affiliated organizations, or those of the publisher, the editors and the reviewers. Any product that may be evaluated in this article, or claim that may be made by its manufacturer, is not guaranteed or endorsed by the publisher.

